# Emotional Experience and Type of Communication in Oncological Children and Their Mothers: Hearing Their Testimonies Through Interviews

**DOI:** 10.3389/fpsyg.2022.834312

**Published:** 2022-05-24

**Authors:** Paula Barrios, Ileana Enesco, Elena Varea

**Affiliations:** Departamento de Investigación y Psicología en Educación, Complutense University of Madrid, Madrid, Spain

**Keywords:** oncology, children, mothers, emotions, communication, interviews

## Abstract

The emotional experience and the type of communication about cancer within the family are important factors for successful coping with pediatric oncology. The main purpose is to study mother’s and children’s emotional experiences concerning cancer, whether they communicate openly about the disease, and relationships between the type of communication and the different emotions expressed by the children. Fifty-two cancer patients aged 6–14 years and their mothers were interviewed in separate sessions about the two central themes of the study: emotional experiences and type of communication. Analyses of response categories were performed to subsequently compare the age-groups and the mother–child responses. According to the results, mothers expressed emotions such as fear, sadness, or anxiety, while children report sadness, pain, but also happiness. Significant positive correlations were observed between mothers’ sadness and older children’s sadness, mothers’ anxiety and children’s fear, and mothers’ anxiety and children’s happiness. Regarding communication type, mothers tend to hide information about the disease from younger children and to provide direct information to the older children. Children usually prefer to communicate their concerns to parents; however, children whose mothers convey anxiety are more likely to prefer to communicate with others. These results support the idea that parents should talk honestly with their children, explaining their illness in an age-appropriate way, and encouraging them to share their emotional experiences. Further studies are needed from a developmental perspective to understand the disease management of children and families.

## Introduction

The incidence of cancer during childhood has grown in the past decade across the world and, nowadays, is the first cause of death by disease among children in Western countries. Although most of the oncological children survive cancer (5-year cancer survival exceeds 80% of the diagnosed; Erdmann et al., 2020), the illness process entails long treatments and painful procedures with high levels of stress and psychological discomfort (Vacik et al., 2001). Apart from the impact the disease has on the organization of family life and on school adjustment, one important source of stress for sick children is the uncertainty about their condition, particularly when they lack any understanding of their illness and people around them do not talk about it. However, omitting information about what is really happening to the children does not protect them from anxiety and distress (Bearison and Granowetter, 2012); on the contrary, maintaining the children in a supposed condition of “ignorance” can lead them to elaborate (or confirm) false ideas about what caused the disease and to what extent some of their past behaviors triggered it (Eiser and Havermans, 1992; Enesco, 2009). Cognitions and emotions of the child with cancer remain little known to professionals and also to many parents. The main objective of this study is to deepen the knowledge of the emotional experiences of cancer from the perspective of both the oncological children and their mothers. We also explore the degree and quality of family communication since the child’s cancer diagnosis and during the process of illness.

Most pediatric oncologists are aware that, at the time of diagnosis, the family may react with a variety of emotions, ranging from disbelief or denial, to anger or despair. Then, during the disease treatment period, which can last from months to years, the family faces unexpected and tough challenges in their daily routines, while dealing with uncertainty and fear of the child’s death (Compas et al., 2012). In such distressful conditions, some family members may suffer helplessness and depression, requiring psychosocial support to protect them from dysfunction, and to help the child face and manage the treatment process (Sloper, 2000; Pai et al., 2007; Hammer et al., 2015; Steele et al., 2015; Brand et al., 2017). Indeed, the child’s quality of life during the disease depends to a great extent on the way family cope with the psychological burden that comes with illness and treatment.

Regarding the children with cancer, there are few studies about their actual experiences, feelings, and cognitions. Most of the information about these issues come from their parents, *via* questionnaires or structured interviews in which they are asked to answer how they think their child is dealing with the disease, or in other terms, how they interpret their child’s behaviors. Some of these studies find that, irrespective of their developmental stage, most children and adolescents have great difficulties to cope with the stressful experiences associated with cancer, showing symptoms of anxiety and or depression (Kupst and Patenaude, 2016; Brand et al., 2017). Compas et al. (2014) analyzed the reports of mothers and fathers about their children’s coping strategies together with measures of children’s emotional distress, and they found some interesting results: anxiety was positively related to disengagement coping strategies (avoidance or denial), whereas negatively related to coping strategies (acceptance and cognitive reappraisal). Anxiety has also been found to relate with a perception of low control over the illness. Moreover, self-esteem is the aspect of identity that suffers most from the disease, and in general, children and adolescents with cancer have to re-adjust their self-perception while coping with the pressure of maintaining a proper school functioning and social competence (Stam et al., 2001).

Other studies have found developmental differences regarding the impact of cancer throughout childhood and adolescence. During childhood and school age (6–10 years), the disruption on school and daily activities can foster a sense of inferiority in the child and, in some cases, feelings of guilt or shame. On the cognitive side, children with cancer may show a better reasoning about cancer and a deeper understanding of death, in addition to more technical information (e.g., side effects of treatments). However, as they become aware of their unfair condition disrupting their normal live, they are likely to develop aversion to the medicine procedures (Brand et al., 2017). Later in adolescence, they may find it harder to cope with their disease as they become increasingly aware of the implications of cancer and the risk of death. Older children cancer patients have a growing desire to develop their own identity beyond cancer and to have a normal and independent life, but they realize that it could be chimerical. Furthermore, maintaining their social relationships can be difficult, making them feel incompetent, frustrated, self-excluded, and lonely (Barlow and Ellard, 2006; Morgan et al., 2010). Despite this, adolescents can improve their sense of efficacy and resilience if they are given the opportunity to participate in the processes of decision making (e.g., outpatient or inpatient treatment) based on honest and truthful information (Stuber et al., 1996; Phipps, 2007; Rosenberg et al., 2014).

In the flow of information, from doctors to parents to the child, it is common for parents to reinforce the opinion that, before adolescence, children are not able to understand their condition; therefore, parents may tend to downplay the importance of what children say and their need to know, with the well-intentioned purpose of protecting them from the harsh reality (Sartain et al., 2000). However, recent studies show that even with young children, giving them age-appropriate but honest information and taking their opinions into account for some decisions fosters their trust in doctors and their commitment to treatment (Thompson and Young-Saleme, 2015). Studies with cancer survivors of childhood cancer reveal that many of them would have preferred to be given honest information including details on the side effects of the illness (Gianinazzi et al., 2014). However, although most children have the desire to know and understand (Bearison, 1991; Enesco, 2009), it is also known that, at all ages, there are individual differences in tolerance to negative information, capability of understanding (Eiser, 1989), and the desire to participate in the decisions (Brand et al., 2017). Some patients may need to adopt a disengagement coping strategy (Compas et al., 2014) for a period of time, and parents should seek a balance between respect for the child and appropriate follow-up treatment. Therefore, it is necessary to take into account all these variables to decide how and when parents should give that information to their children (Stein et al., 2019).

There has been little research that considers children’s narratives as representative of their experiences with the disease. This may be due to the traditional view of children as passive beings during the illness process (Gibson et al., 2010), but also to the cost of doing qualitative research based on interviews that requires a large investment of time in the collection and analysis of data. Despite this, there is no better way to know what children feel and think than to talk and to listen to them (Bearison, 1991). And the same is true for parents: a narrative about their experience will give us more and richer information than their answers to a questionnaire (Sartain et al., 2000).

In Spain, one of the few studies that addressed children’s conceptions and understanding of cancer, using a qualitative methodology, was Enesco (2009). She implemented a semi-structured interview to explore the conceptions that healthy and oncological children (6–14-year-olds) had about cancer. She found that children’s comprehension of cancer was associated with age, irrespective of the ill-health condition. In particular, in both groups the false beliefs around the causes of cancer were more likely to appear among children under 9 than in later ages. The ill-health condition had little effect on most of the conceptual issues (e.g., those related to causes), except for the prognosis. Whereas many of the healthy participants aged 10–14 perceived cancer as an extreme severe disease and mentioned the risk of death, virtually none of the sick participants said that the prognosis of cancer could be fatal. Certainly, these findings reveal the intricate relationships between cognition and emotion. Unfortunately, the Blinded’s study includes little information about the emotional side, that is, about what children expressed regarding the emotions during the illness process and treatment.

The purpose of this study is to enhance knowledge of the emotional aspects of the experience of cancer during childhood from the perspective of both the ill child and the mother. We want to disclose how the family coped emotionally with the illness at the time of diagnosis and how the mothers, in particular, explained to the child his or her disease. We explore different relations between the mothers’ and children’s emotional coping with cancer and the type of communication in the family. We also investigate the relations between the emotions conveyed by the mother and the children preference or not to share their feelings with their mother. All these objectives are approached from a developmental perspective, comparing the two age-groups.

## Materials and Methods

### Design

Our research design is a qualitative descriptive study based on semi-structured interviews, with a cross-sectional temporal design. The participants (oncological children and their mothers) were chosen by convenience sampling. The type of data obtained allowed for problem-driven content analysis and inductive reasoning approach (Krippendorff, 2013; Patton, 2015). The resulting categories subsequently allowed for quantitative and comparative analyses between groups (see below Data coding and analysis).

### Participants

The participants were oncological patients from two hospitals of Madrid specialized in childhood cancer treatment (Niño Jesús and La Paz Hospitals). A total number of 52 oncological children were selected, aged from 6 to 14 years, as well as their 52 mothers. The decision to interview the mothers was for convenience since, in most cases, it was the mothers who accompanied the child for treatment or revision. Moreover, all the mothers participating in the present research were the primary caregivers at home.

All the children met the inclusion criteria of not suffering any serious cognitive or physical side effects of either treatment or surgeries. The medical staff of each hospital actively helped in the selection of the sample, since they knew the individual conditions of the children. We interviewed children who had been diagnosed at least 3 months ago, excluding those who had just received their diagnosis. According to the medical staff, newly diagnosed children may not yet have assimilated their situation, or may even be unaware of it, while those diagnosed three or more months earlier already have experience with the disease process and treatment.

The study was approved by the Ethical Review Board at both hospitals, and it was conducted in accordance with the Declaration of Helsinki regarding research ethics (World Medical Association, 2013). The mothers gave a written consent to be interviewed themselves and their children, and verbal consent was obtained from the children. All participants were informed that they could withdraw the interview at any time if they wished. During the interview, the researcher was very attentive to any negative emotional reaction from the participant, reminding, if necessary, their right to stop the interview. Only one mother, who requested to be present during her son’s interview, asked to interrupt the interview when it came to talk about the prognosis of the character’s disease (see procedure). As this participant did not complete the interview, he was not included into the final sample.

### Procedure

We implemented two semi-structured interviews with open-ended questions aimed at understanding both the child’s and the mothers’ subjective experiences with cancer (see Table 1 for a description of the interviews).

**Table 1:** Main questions of the semi-structured interviews with mothers and children.

Target group	General questions (specific probe questions are not included here)
Mothers	When did you know that your son/ daughter was ill? (The central questions of this interview were raised after asking the mother: *when* did she learn that her son/daughter had the disease, *who* told her, and who was present at the time). Which were the main emotions you felt at that moment? And since then, how have you been experiencing your son/daughter’s illness? (We encourage the mother to express all the emotions she remembered feeling…). How did it affect family? (This very general question seeks to delve into the emotional experiences of the mother in the family context, in case she had not been able to express them before. It also gives rise to the following question on communication with the sick child). Did you tell your son/ daughter that he/ she was ill? Why (Yes or not)? (To find out if the information given to the child was forthright, nuanced, distorted, or simply hidden, and the reasons for such a decision).
Children	After having shown the pictures to the participant and asked about what happened in the scenes (Identification of the illness, severity, attribution of causes, etc.), the interview addressed the feelings and emotions of the character: How do you think he (The character) feels at the hospital? (We encourage the children to express all the emotions attributed to the character, as well as any reference to their own situation and feelings). What do you think makes him feel worse? Try to figure out all the things that can make him feel bad… What do you think he could do to feel better? What could others do to make him feel better? Try to figure out all the things that can make him feel good… Do you think he would like to communicate with someone about his feelings? Why (Yes or no)? If yes: To whom? Who do you think he would like to talk to? Anyone else? (The child is invited to express any need or desire to talk to others about their condition and feelings. In an indirect way, we encourage them to think of specific people with whom they would like to communicate).

The child’s interview was partially based on the long interview developed by Domínguez-Ferri (Enesco, 2009), including additional questions about the emotions linked to the experience at the hospital and at home, their need, or not, to communicate with the family or others, and talk about it, etc. A pilot study allowed testing and adapting the questions particularly with the younger children. The child’s interview began with a brief story illustrating the hospital stay of a group of children. One of them, the main character (a sick child in the hospital), goes through different medical procedures and other daily situations at the hospital. This starting point made the conversation with the child much easier: in all cases, the children began by talking about the character and ended up talking about themselves. This way of approaching the disease has been shown to be more sensitive and respectful of the sick children than asking them directly about their condition (Enesco, 2009).

The mothers’ interview assessed specific aspects about their emotional reaction to the illness at the time of initial diagnosis, the degree and quality of communication with the child regarding his or her disease (e.g., unveiling or not the real diagnosis), and experiences during treatment.

To confirm the information given by the medical staff about the child’s disease (type of cancer, type and duration of the treatment, time under hospitalization), the mothers were first presented with a brief questionnaire about all these aspects.

The interviews were conducted by the first author in a quiet room at the hospital, and they were audio-recorded for the subsequent verbatim transcription. The interviewer was experienced in communicating with cancer patients since she had volunteered for psychological care of children with cancer. The length of the children’s interviews varied considerably depending on the participant’s age, with an average length of 10 min (8–16 min. range). The mothers’ interviews lasted an average length of 20 min (12–40 min. range).

### The Interviews With Mother and Children

### Data Coding and Analysis

According to the nature of the data collected, a problem-driven content analysis and inductive reasoning were performed (Krippendorff, 2013; Patton, 2015). This analysis allows for the identification of units of meaning in the participant’s responses throughout the interview. As reported in Anguera et al. (2018), “narratives are an excellent vehicle for studying everyday life,” and integrating quantitative and qualitative data we can obtain a more complex and complete methodological framework.

The coding process followed different phases. Firstly, all the interviews were literally transcribed. Secondly, we established high-level categories (Patton, 2015) that correspond to the broad concepts or topics addressed in the interview: emotional experience and communication of the illness to the child (see Table 2). Thirdly, through a careful interpretative process of the participants’ responses, we defined a set of lower-level categories (León and Montero, 2015; e.g., 1.1 Fear, 1.2 Anger or Rage, etc., within the “1. Emotions” higher category, see Table 2). Establishing high-level and low-level categories of the narratives provides order and hierarchy among the answers of the participants facilitating the posterior analysis. Fourthly, the answers given by the participants were coded within the different hierarchical categories. This allows the frequencies of specific interview topics to be quantified so that comparisons and correlations can be made with other measurement techniques (Kvale, 2008).

**Table 2:** Coding categories of the semi-structured interviews to the family and the children.

Sample group	Higher-level categories	Lower-level categories
A. Mothers	Emotions	1.1 Fear 1.2 Anger 1.3 Sadness 1.4 Frustration/Impotence 1.5 Anxiety 1.6 Guilt
	Communication of the illness to the child	2.1 Forthright information 2.2 Nuanced/distorted information 2.3 No information at all
B. Oncological children group	Emotions	1.1 Fear 1.2 Anger 1.3 Sadness 1.5 Pain 1.6 Boredom 1.7 Loneliness 1.8 Happiness
	Factors related to negative emotions during hospitalization	2.1 Physical effects (pain, queasiness, hair loss) 2.2 Absence of family 2.3 Not attending school 2.4 Lack of social relationships 2.5 Negative thoughts 2.6 Hospital environment 2.7 No answer
	Activities that improve the emotional state	3.1 Playing games 3.2 Family visits 3.3 Social relationships 3.4 Be able to adapt to the hospital environment 3.5 Attending the hospital school
	Communication about their feelings	4.1 Yes: need to communicate 4.1.1 Family members 4.1.2 Friends 4.1.3 Medical staff 4.1.4 Psychologists 4.1.5 Other oncological children 4.2 No need to communicate

All the emotions mentioned by mothers and children have been listed. As can be seen, mothers and children only coincided in three of these emotions: fear, anger, and sadness.

In order to ensure a rigorous coding process, we created a coding dictionary that included a precise definition of each high- and low-level category with examples illustrating types of responses. This ensured multiple perspectives on the data, as suggested by Corbin and Strauss (2008), to increase creativity in the analysis while also decreasing personal bias. An inter-judge assessment was carried out by two independent coders, expert in content analyses (the first two authors of the paper), reaching an agreement in 90% of the coding among the different categories. In cases of doubt or disagreement, discussions were held between the two authors to solve them and reach a conceptual-based consensus on the coding.

For analysis purposes, we divided the sample into two age-groups: 6–9 years of age (young children, from now on) and 10–14 years of age (older children, from now on). This decision was taken considering that previous studies have shown that, from the age of about 10, cognitions about illness and health processes become more articulated and realistic (Bibace and Walsh, 1983; Potter and Roberts, 1984; Enesco, 2009).

We present the results around the two general axes of the interview: the emotional experiences of children and mothers, and the type of communication in the family regarding the child’s illness. All analyses were performed using SPSS version 27.0.

For the emotional experiences, we begin by presenting a descriptive analysis of the prevalence (%) of the emotions conveyed by mothers and children. Second, to check for possible differences in the emotions mentioned by the different groups (mothers, younger, and older children), we perform Pearson’s Chi-square tests. Third, to explore possible relationships between the emotions reported by mothers and those reported by their own children, we use McNemar’s tests. Finally, we present descriptive analyses of the (positive and negative) experiences children reported having during hospitalization.

Regarding mother–child communication, we first present a descriptive analysis of the degree of information given to the child (straight, nuanced or hidden information). Second, to assess possible relations between degree of information and the child’s age, we use Pearson’s Chi-square test. Third, to explore any relationship between degree of information given by the mother to the child and his or her emotions related to the illness experience, we use McNemar’s test. We also analyze children’s responses to the question about people to whom they would like to communicate their experiences. By McNemar’s tests, we perform different analyses relating these responses to those provided by mothers regarding their own emotions.

## Results

### Emotions in Mothers and Children

Before describing the results of this section, it should be remembered that the questions about emotions were different for mothers and children. The mothers were requested to talk about their own emotions and feelings at the moment of diagnosis and during the child’s illness process. The children were asked to talk about the emotions they attributed to the character (the sick child in the hospital). During the interview, however, the children often referred to their own experience and continued to talk about it. In any case, the analyses were performed on all the child’s responses around emotions, whether attributed to the character or to himself or herself.

Table 3 shows the prevalence of the emotional experience of both mothers and children. As can be seen, the emotions most often mentioned by the mothers were fear and sadness, followed by impotence, anxiety, and anger. Only three mothers also mentioned the feeling of guilt, related to past decisions (not having anticipated that the child was showing symptoms of sickness) or to her own mood or the way of coping with her child’s illness. On the other hand, among the children, the emotion of sadness was the most frequently named (virtually all 10–14-year-olds and two-thirds of the 6–9-year-olds referred to feel sadness). Other negative emotions named by children were boredom, pain, and anger. Several children of both age-groups mentioned the emotion of happiness when describing the feelings while staying at the hospital (see below for further details on this).

**Table 3:** Emotions mentioned by mothers and children (6–9 and 10–14 years) during cancer experience.

	Children						Mothers					
	6–9 Years (*n* = 24)		10–14 Years (*n* = 28)		Total (*n* = 52)		6–9 Years (*n* = 24)		10–14 Years (*n* = 28)		Total (*n* = 52)	
	*n*	%	*n*	%	*n*	%	*n*	%	*n*	%	*n*	%
Fear	1	4,2	6	21,4	7	13,5	14	58,3	14	50,0	28	53,8
Anger/rage	4	16,7	8	28,6	12	23,1	8	33,3	5	17,9	13	25,0
Sadness	16	66,7	27	96,4	43	82,7	9	37,5	14	50,0	23	44,2
Frustration/impotence							8	33,3	11	39,3	19	36,5
Anxiety							8	33,3	9	32,1	17	32,7
Guilt							1	4,2	2	7,1	3	5,8
Happiness	14	58,3	15	53,6	29	55,8						
Pain	9	37,5	8	28,6	17	32,7						
Boredom	9	37,5	9	32,1	18	34,6						
Loneliness	1	4,2	2	7,1	3	5,8						
Shame	2	8,3	0	0,0	2	3,8						

The total number of answers might be superior to the number of participants as children and mothers could mention different sub-categories.

Secondly, with regard to differences by age-group, younger children more commonly mentioned emotions such as physical pain and boredom (37.5% the younger children and around 30% the older children), while older children reported greater sadness (96.4%) than younger children (66.7%), and mentioned the emotions of anger slightly more (28.6%) than did the youngers (16.7%). Three children referred to loneliness (one from the younger group and two from the older group), and only two young children mentioned the emotion of shame related to the fact of being bald or hair loss. However, none of the above-mentioned emotions differed significantly between younger and older children (Chi-square tests, *p* > 0.05), except sadness: older children were more likely to mention it than younger ones (Chi-square test, *χ*^2^ (1, *N* = 52) = 7.998, *p* < 0.005).

Comparing now the emotions reported by mothers and children (Table 3), we can see that fear, impotence, and anxiety were mentioned almost exclusively by mothers: out of 52 mothers, 28 referred to fear, 19 to impotence, and 17 to anxiety; none of the children mentioned impotence or anxiety, and only six children (five of them 10–14-year-olds) referred to fear. Instead, both mothers and children alluded to sadness in not negligible proportions. A McNemar test analysis comparing the mother group with the two age-groups (younger and older children) revealed two significant relations between emotions reported by mothers and children. On the one hand, children whose mothers mentioned the emotion of sadness were more likely to report this emotion, but this was significant only for the group of older children (McNemar test, *p* = 0.001). On the other hand, children whose mothers reported anxiety were more likely to report fear (McNemar test, *p* = 0.041).

### The Emotion of Happiness

Intriguingly, an emotion often cited by children, never by mothers, was happiness. Thus, 58.3% of the younger children and 53.6% of the older children attributed some kind of joy or happiness (estar contento, alegre, in Spanish) to the character, during his stay in the hospital, with no significant differences between age-groups in this category (Chi-square tests, *p* > 0.05). Although this attribution seems very disconcerting, the explanations given by children provided a way to make sense of it. For example, a child aged 9 said “he feels happy because they are helping him and he will be better.” A child aged 12 years explained: “you have to be happy because you have no other choice, you will not live-in bitterness, I mean, you cannot stay in the hospital and also be worried, I think he is happy, positive, having fun with the other friends, with his roommate.” Another child aged 14 said: “I think he is happy but at the same time he is a little sad. A little sad because he knows his situation and happy because he has to think positive and so he will recover.” This type of justification was indeed more frequent among the older children than the younger children, but overall, it indicates that they are talking about joy or happiness as a form of positive thinking that would be necessary for healing, or at least increasing wellbeing during hospitalization. Because of the unexpectedness of this finding, we examined whether there were any relationships between the children reporting the emotion of happiness and their mothers reported emotions. We found only one significant positive relation between happiness (children), and anxiety (mothers; McNemar test, *p* = 0.036).

### What Makes the Mood Worse or Better During Hospitalization?

The interview included questions about factors that could worsen or improve the patient’s mood during treatment or and hospitalization. Tables 4 and 5 show the response categories for factors related to negative and with positive experiences, respectively.

**Table 4:** Factors related to negative emotions during hospitalization.

	Children					
	6–9 Years (*n* = 24)		10–14 Years (*n* = 28)		Total (*N* = 52)	
	*n*	%	*n*	%	*n*	%
Absence of family	2	8.3	5	17.9	7	13.5
Not attending school	1	4.2	5	17.9	6	11.5
Lack of social relationships	2	8.3	10	35.7	12	23.1
Negative thoughts			12	42.9	12	23.1
Inactivity/hospital environment	10	41.7	14	50.0	24	42.3

The total number of answers might be superior to the number of participants as children and mothers could mention different sub-categories.

**Table 5:** Activities reported by children related to emotional wellbeing during hospitalization.

	Children					
	6–9 Years (*n* = 24)		10–14 Years (*n* = 28)		Total (*N* = 52)	
	*n*	%	*n*	%	*n*	%
Playing games	14	58.3	20	71.4	34	65.4
Visits	8	33.3	11	39.3	19	36.5
Social relationships at the hospital	4	16.7	5	17.9	9	17.3
Adaptation to the hospital environment			2	7.1	2	3.8
Attend to the hospital school			3	10.7	3	5.8

The total number of answers might be superior to the number of participants as children and mothers could mention different sub-categories.

For the factors related to negative experiences, two were frequently mentioned by the participants of both age-groups: the physical sensations and effects of the treatment (pain, queasiness or hair loss, 50% and 37% of younger and older children, respectively), and inactivity or the hospital environment itself (42% and 43% of younger and older children, respectively; e.g., “the things they do to him in the hospital really hurt”; “he feels pretty bad because he is bald,” “having to be in the hospital doing nothing, and not being able to leave the hospital”). The absence of family (18% vs. 8% of younger) and not attending the school (18% vs. 4% younger) were mentioned mainly by the older children as well as the lack of social relationships (37% vs. 8% of younger children) with significant differences between ages only in this latter [Chi-square test, *χ*^2^ (1, *N* = 52) =5.548; *p* < 0.05]. But the most acute difference between age-groups was the reference to negative thoughts about the illness. Nearly half of the 10–14-year-olds (43%), but none of the younger children, talked about having negative thoughts related to fear of a worsening of the disease. For example, a 12-year-old child said: “maybe you think… and you are afraid they will not tell you when you are going to leave the hospital”; and another 14-year-old child said: “scared to think that you are not going to be healed.”

For the factors related to positive experiences during hospitalization that could improve the child’s emotional state (Table 5), the two most common answers were playing games with other children (58% and 71%, younger and older) and visits of family or friends to the hospital (33% and 39%, younger and older). Nevertheless, none of the above-mentioned factors differed significantly between younger and older children (Chi-square tests, *p* > 0.05).

### Communication in the Family

Regarding the communication and information given to the child, as seen on the Table 6, the mothers’ responses were grouped into three exclusive categories: direct honest information; nuanced or distorted information; and no information at all. The direct information category included testimonies such as “we explained her all the information about her disease, and as soon as she had questions or doubts, we answered her”; “we never hid the truth… that he had a cancer,” or “we told him the diagnosis and later, we talked about the treatment and the surgery.” The nuanced or distorted information category included testimonies such as “I told him that he had bronchitis, because he is young and he could not understand what is cancer” or “we do not talk about cancer, she knows that she has cancer but not really what it is, we use to take away the importance of it.” Some mothers said that they partially explained the diagnosis but not the medical procedure in order to preserve the child from fear and anxiety. Finally, in the no information category the mothers’ testimonies were clear-cut: they asserted to have omitted all information from the child, both the real diagnosis and the medical procedures to come. In its pure form, this last category was infrequent since most testimonies referred to having given the child a distorted or nuanced information about his or her symptoms and treatment (a total silence about the disease is, indeed, quite improbable to occur when it concerns cancer). Therefore, we have merged the two last categories (nuanced or distorted and no information) into one category for analysis purposes.

**Table 6:** Communication of illness from mothers to children depending on age-group.

	Children					
	6–9 Years (*n* = 24)		10–14 Years (*n* = 28)		Total (*N* = 52)	
	*n*	%	*n*	%	*n*	%
Nuanced or hidden information	15	62.5	5	17.8	20	38.4
Forthright information	9	37.5	23	82.1	32	61.5

The results showed that most of the older children (82.1%) had received forthright and truthful information about their illness, while only some of the younger children (37%) received truthful information, with significant differences between ages in this regard [Chi-square test, *χ*^2^ (1, *N* = 52) = 47.763; *p* < 0.001].

We explored whether the degree of information given to the child was related to any of the emotions reported by him or her (see Table 1) and found only one relation with the child’s emotion of fear: children who had received truthful information were significantly less likely to mention fear (McNemar tests, *p* = 0.011). However, it is worth noting that the total number of children who reported fear was very small.

When the children were asked whether they would feel the need to talk to others about their illness, virtually all said yes. It is important to note that this need was expressed also by children whose families had not given them honest information about their illness. Regarding the question of who they would like to talk to, most of them reported their preference to communicate with parents and close relatives (family members, 54% and 79%, younger and older children), followed by friends (33% and 36%, younger and older children), and medical staff (21% and 39%, younger and older children; Table 7). Remarkably, the psychologists were mentioned almost exclusively by the older children (54%).

**Table 7:** People to whom children prefer to communicate their emotional state.

	Children					
	6–9 Years (*n* = 24)		10–14 Years (*n* = 28)		Total (*N* = 52)	
	*n*	%	*n*	%	*n*	%
Family members	13	54.2	22	78.6	35	67.3
Friends	8	33.3	10	35.7	18	34.6
Medical staff	5	20.8	11	39.3	16	30.8
Psychologists	2	8.3	15	53.6	17	32.7
Other oncological children	5	20.8	3	10.7	8	15.4

The total number of answers might be superior to the number of participants as children and mothers could mention different sub-categories.

We explored whether children willingness to communicate with parents was related to any of the emotions reported by their mothers. We found that mothers reporting (or not) anxiety about the child’s illness was related to children’s preferences for communication. In particular, children whose mothers expressed anxiety were more willing to communicate their own emotions to other people rather than to the family; correlatively, children whose mothers did not express anxiety were more willing to communicate with parents (McNemar test *p* = 0.003).

## Discussion

The present study explored two main aspects related to the experience of cancer in oncological children and their mothers: their own emotions related to the disease and the communication in the family regarding the child’s illness. We explored several relationships between these two aspects comparing the two age-groups on the different topics assessed.

Mothers and children reported a variety of emotions related to the experience of cancer, three of which were common to both groups (sadness, anger, and fear) but in different proportions. More than half of the mothers talked about fear as the most prevalent emotion at the time of diagnosis and thereafter, while only very few children (six out of 52) explicitly mentioned fear of events related to the course of the disease, such as pain or uncertainty about healing. A previous study with oncological and healthy children (Enesco, 2009) showed a notable difference between these two groups in their prognosis of cancer disease: the ill children had a rather optimistic view of the course of the disease, and they rarely mentioned death, while the healthy children had a fairly pessimistic perspective about the outcome of the disease, often mentioning the risk of death (particularly, the older children). Our findings are quite in line with those of Blinded, suggesting that oncological children tend to “avoid” as much as possible a negative perspective on their situation. However, it does not imply their fears have disappeared. In fact, as the interview progressed and children were asked about what could cause a negative mood during hospitalization, around half of the older participants (although none of the younger ones) elaborated on their dark thoughts about the threat of not being healed.

The results on the emotion of sadness are consistent with the above idea. Both mothers and children talked about sadness, but particularly the older children, who (all but one) described sadness as the core emotion of the cancer experience. Past research has shown that as children get older, they develop more elaborate and complex ideas about health and illness (Carey, 1985; Myant and Williams, 2005; Inagaki and Hatano, 2006; Zhu et al., 2009). Among older children with cancer, this improvement in understanding is paralleled by greater awareness and concern about their own condition, its severity and prognosis. Thus, it is possible that the high prevalence of sadness together with the negative thoughts mentioned by the older children may reflect an acute concern about the prognosis and, as a consequence, some kind of depressive mood. Intriguingly, however, neither the mothers nor the children used the term “depression” at any time. We may wonder whether, when mothers and children speak of sadness, they could also be referring to some form of depression. In other words, “sadness” might be a colloquial way of referring to depression, or a euphemism that circumvents the more serious term of depression. On the other hand, the unexpected emotion of “happiness,” mentioned by more than half of all children, gives us some important clues about the emotional processing and coping strategies of oncological children. It has been observed that, in general, families perceive the severity of the disease more intensely and realistically (or pessimistically) than their children, who are more prone to express some positive expectations (Davison et al., 1992). As we pointed out when describing this result, the children may be talking about happiness as a positive attitude that favors curing. This brings us to a viewpoint with increasing prevalence in the literature since the late 1990s: the role of positive thinking in healing. According to some authors, there is a high moral and psychological pressure on several cancer patients to practice positive thinking about their disease (Raeve, 1997), and although this pressure can encourage hope and the search for “supportive relationships” (O’Baugh et al., 2008), it can also have aversive emotional consequences for patients. As Rittenberg (1995) stated, some patients can feel forced to accept a positive mental attitude by their medical professionals, not permitted to face their reality freely, and therefore adopting a “repressive adaptation style” to face their illness (Phipps, 2007). Our data do not allow us to affirm that the children who mentioned happiness were adopting this repressive style, but it is a plausible hypothesis. As previously discussed, the expression of positive emotions that are not expected in a hospital environment may occur with greater intensity in children with anxious mothers as an adaptive attitude to reduce the general anxiogenic level in the family. This is consistent as well with the notion of positive reappraisal, defined as a cognitive emotional regulation strategy (Carver et al., 1989; Garnefski et al., 2001). Positive reappraisal is a form of positive thinking or optimism in the face of negative events, which helps turning attention to the positive sides of those events. In line with previous research that found a negative association between positive reappraisal and anxiety (Carver et al., 1989; Garnefski et al., 2001), we can venture that the emotion of happiness mentioned by children would be part of a coping strategy to regulate not only their own emotions but also the emotional states of others, thus trying to reduce their mothers’ anxiety. Further research regarding positive beliefs and thoughts in cancer patients will be necessary to obtain a better understanding of coping strategies and emotional experience.

Hospitalizations during the treatment of cancer are common, and many hospitalized children reports this experience as a traumatic event (Le Brocque et al., 2010). At present, there is a widespread awareness of the need to humanize the experience of the hospitalized child, and many oncological centers offer leisure and gaming activities in order to reduce the impact of hospitalization on children. In our study, children and preadolescents were given the opportunity to explain what affected most their (negative) mood and thoughts, and what could be done to improve their emotional state. For many of them, the worst part of the experience was the hospital environment itself (not providing activity and distraction, but also because of the painful medical treatments). Interestingly, the older children gave more importance to the limited social relations during hospitalization (particularly, the absence of schoolmates) than the younger children, probably due to the greater need of older children to maintain their social networks with peers, beyond the family. Moreover, only the older children reported the presence of negative thoughts and talked about concern and fear during hospitalization. As mentioned before, this is indeed coherent with the increasing consciousness of death, from preadolescence, and the fear associated with uncertainty. It is not surprising, therefore, that several older children claimed the need to also talk to psychologists, as a support to deal with their dark thoughts.

Regarding the communication of mother and child, our results revealed that their degree and quality varied between families, but particularly depending on the age of the child, consistently with previous findings (Sartain et al., 2000; Stenman et al., 2019). Most of the older children’s mothers reported to have an honest communication with their children regarding their disease and treatment. In contrast, the mothers of young children commonly acknowledged to have kept their children away from important decisions and to have concealed or disguised the diagnosis and treatment details. This result is not surprising to the extent that it converges with the representation of young children as passive and immature subjects that are not prepared to understand their situation (Claflin and Barbarin, 1991; Sartain et al., 2000). Unfortunately, there are few systematic researches regarding the relationships between a child’s comprehension of cancer and their subjective experience of the illness, emotional regulation, and coping strategies (Méndez et al., 2004). Nevertheless, we do know that listening to children and answering their questions foster mutual communication based on trust, which is essential to face the long and painful process of the disease. This idea is not new among researchers involved with oncological children. At least since the 1990s, several authors have been insisting on the need to respond to children’s questions and concerns with age-appropriate explanations (Bearison, 1991; Eiser and Havermans, 1992; Kenyon, 2001; Wilkins and Woodgate, 2005; Thastum et al., 2008). The benefits of such honest communication have been observed in different areas: It reduces the child’s fears and uncertainty, increases compliance with treatment, and improves the acquisition of healthy coping strategies (Stein et al., 2019).

## Strengths and Limitations

One of the strengths of this study has been to have interviewed children with cancer as well as their mothers since there are not many qualitative studies with oncological patients that include family members. The use of interviews allowed us to obtain rich information about the children’s and mothers’ subjective experiences regarding the disease. The mothers gave substantial information about their own experience since the time of diagnosis and during the child’s treatment. Some mothers also talked about other family members but without elaborating in sufficient detail, given that the interview focused primarily on their own experiences. Therefore, this study would certainly be richer if we had also interviewed the fathers or other family members whose perspective, emotional experiences, and coping styles might be different from that of the mothers.

Other limitations of the present work refer to variables that have not been controlled and whose study could yield interesting data. For example, how does the number of hospitalizations affect patients’ emotions and coping strategies? Do these emotions and strategies change depending on the time since diagnosis? It is important to highlight the need for studies specifically designed to assess whether time since diagnosis has an influence on aspects such as the patients’ representation of their disease, their copying strategies, and emotions associated with their experience.

Regarding methodological aspects, it would be of great interest to complement the verbal information of the interviews with visual information of the participant’s emotional facial reactions. Techniques such as those used in Morales-Sánchez et al. (2020) would be appropriate for taking measurements of the participants’ facial reactions during the course of the interview, revealing aspects that may have gone unnoticed by the interviewer.

On another line, it is essential to investigate what ideas oncology healthcare professionals have about the emotional needs and cognitive abilities of children, as a starting point for developing training programs for this collective. Children often spend a great deal of time in the hospital interacting with the oncology health staff, so it is necessary to train these caregivers in ways to communicate with children while preserving their psychological wellbeing. In addition, it would be of interest to conduct further studies that may reveal differences between healthy and ill children in relation to some of the research variables.

Finally, we have found some intriguing relationships between mothers’ and children’s emotions that have led us to ask the following question: Is the mother’s anxiety related to the child’s need for positive thinking as an adaptive attitude to reduce the overall anxiogenic level in the family? Our results point in that direction, but further study of this relationship is needed to delve deeper into the emotions of mothers and children with cancer.

## Conclusion

Pediatric cancer is a very challenging diagnosis to cope with emotionally for both children and family, and the type of communication within the family can favor or hinder the coping strategies of family members. Our results revealed some differences in the emotions disclosed by children and mothers and also according to the age of the children. A particularly interesting finding was that several children of all ages spoke of “happiness” in a sense akin to positive reappraisal in the face of adversity, while only older children revealed having dark thoughts about their present and future. Differences were also found in the type of communication in the family, with younger children being more likely to be kept in the dark than older children.

Beyond the results discussed in this work, it is worth noting that, during the interviews, virtually all the children showed a real willingness to talk about their concerns. On this basis, new studies should deepen the question of when and how to talk to children about their disease and help them coping with it. To this end, an important variable to be considered is the child’s developmental level in terms of cognitive and socioemotional capacities. There is no doubt that the experience of illness is very complex and many variables are involved, but whatever the age and level, children cannot be left in uncertainty about what is happening to them by avoiding talking about it.

## Data Availability Statement

The raw data supporting the conclusions of this article will be made available by the authors, without undue reservation.

## Ethics Statement

The studies involving human participants were reviewed and approved by the Hospital Universitario la Paz (Madrid, Spain); Hospital Universitario Niño Jesús (Madrid, Spain). Written informed consent to participate in this study was provided by the participants’ legal guardian/next of kin.

## Author Contributions

PB performed the conceptualization, methodology and protocol design, data collection, data analysis, and writing—review and editing. IE did conceptualization, methodology and protocol design, supervision of data analysis, general supervision, and writing—review and editing. EV was involved in generation and supervision of tables and figures, data analysis, and writing—review and editing. All authors contributed to the article and approved the submitted version.

## Conflict of Interest

The authors declare that the research was conducted in the absence of any commercial or financial relationships that could be construed as a potential conflict of interest.

## Publisher’s Note

All claims expressed in this article are solely those of the authors and do not necessarily represent those of their affiliated organizations, or those of the publisher, the editors and the reviewers. Any product that may be evaluated in this article, or claim that may be made by its manufacturer, is not guaranteed or endorsed by the publisher.
