# Supplementary material for: Emotional Experience and Type of Communication in Oncological Children and Their Mothers: Hearing Their Testimonies Through Interviews
Source: Front Psychol. 2022 May 24;13:834312. doi: 10.3389/fpsyg.2022.834312 (PMC9171433; doi:10.3389/fpsyg.2022.834312)
Supplement: Supplementary file 1 [file Table_1.docx]

**Annexes**

Coding manual

Interview 2: Mothers of children with cancer

| **Preguntas y Categorías primarias** | - **Categorías secundarias** | - **Ejemplos de respuestas** |
| --- | --- | --- |
| 1. **Reacción emocional ante el pronóstico**   *¿Cuándo os enterasteis de que vuestro hijo estaba enfermo?*  *¿Cuál fue vuestra reacción ante ese diagnóstico?*  *¿Qué emociones sentisteis en el momento de diagnóstico?* | 1. Emociones:    1. Miedo    2. Enfado/ rabia    3. Tristeza    4. Frustración/ impotencia    5. Ansiedad/ angustia    6. Culpa | - 1. *“Sentí miedo, susto” “Miedo a que vaya mal, a que ella lo pase mal”*   2. *“Rabia, preguntarte por qué” “Mucha rabia”*   3. *“Dolor, mucho dolor y tristeza” “Nos sentíamos demasiado tristes”*   4. *“Impotencia porque no puedes hacer mucho” “Impotencia porque no la puedes ayudar”*   5. *“Mucha ansiedad y angustia, multiplicado por 1000” “Nervios, muchos nervios y angustia”*   6. *“Culpa por no saber si ha sido algo que he hecho mal” “Típicos sentimientos de culpa porque no lo han visto antes”* |
| 1. **Comunicación con el niño respecto a la enfermedad**   *¿Cómo le comunicasteis que estaba enfermo?* | 1. Comunicación con el niño:    1. Directa y honesta    2. Matizan la enfermedad    3. Ocultan la enfermedad | - 1. *“Y yo la verdad es que la expliqué todo, le dije que tenía una cosa en el cerebro que se tenía que quitar, le dije que muy probablemente se le iba a quedar una parte del cuerpo inmovilizada, pero que con rehabilitación iría mejor. La verdad es que le expliqué mucho” “Se lo contó todo la oncóloga, le dieron un librito en el que le explicaba todo”*   2. *“Le dijimos que tenía un tumor, pero como era tan pequeño no le dijimos la gravedad de lo que tenía” “La verdad es que tampoco llega a entender mucho porque claro es muy pequeño, él sabe que tiene una manchita ahí, que tenemos que venir a hacerse revisiones para que no crezca, pero nada más.”*   3. *“No le hemos dicho nada”*   *“No le dijimos nada, ni le hablamos de quimioterapia ni nada”* |
| 1. **Atribución causal del cáncer**   *¿Cuáles crees que son las causas del cáncer de tu hijo?* | 1. Causas del cáncer infantil    1. No sabe la respuesta    2. Causa externa-religión    3. Causa interna-factores físicos    4. Aleatorio    5. Genético | - 1. *”No lo sé” “Yo no sé si comprendo la enfermedad y las causas”*   2. *“Después de haber tenido un niño en teoría sano y que nos haya pasado esto es que dices ‘Dios en qué momento’ ” “Como te puedo decir, el que está arriba decide”*   3. *“Parecía que tenía anemia al principio y que eso era la causa” “Empezó diciendo que le dolía la boca, y en la revisión, claro, descubrieron el ganglio que luego pasó a ser maligno”*   4. *“Me ha tocado a mí” “No hay un por qué, nos ha tocado y nos ha tocado”*   5. *“Creo que la hermana de mi madre tenía cáncer” “Otros familiares también han tenido cáncer de otro tipo”* |

Interview 2: children with cancer

| **Preguntas y Categorías primarias** | - **Categorías secundarias** | - **Ejemplos de respuestas** |
| --- | --- | --- |
| 1. **Identificación de la enfermedad**   *¿Qué crees que le pasa a Jorge? ¿Por qué?*  *Cuando alguien enferma de eso, ¿Qué crees que le pasa?* | 1. Identificación de la enfermedad:    1. Cáncer    2. Otras enfermedades | - 1. *“Tal vez esté enfermo de cáncer” “Tiene leucemia o un tumor”*   2. *“Le duele la garganta” “Está malo y tiene fiebre, tiene dolor de tripa”* |
| 1. **Atribución causal del cáncer**   *Te voy a decir lo que le pasa a Jorge. Jorge tiene cáncer. ¿Sabes qué es el cáncer?*  *Cuando alguien enferma de eso, ¿Qué crees que le pasa?*  *¿Por qué crees que Jorge ha enfermado?*  *¿Crees que los demás niños han enfermado por lo mismo que Jorge?* | 1. Causas de la enfermedad:    1. Anecdótica/ sin respuesta    2. Conducta imprudente puntual/ Higiene    3. Alimentación    4. Golpe    5. Contagio    6. Virus-Bichos    7. Predisposición    8. Contaminación relacionada con el medio    9. Aleatoria | - 1. *“No lo sé” “Pues que se le haya caído el pelo y se lo hayan cortado”*   2. *“Que habrá comido algo del suelo” “Que no se quería poner el abrigo y se constipó”*   3. *“Pues que han comido mal muchas veces” “Por la alimentación, por no comer bien, que no se alimentan bien”*   4. *“Que se ha dado un golpe muy fuerte” “Porque se habrán caído o se habrán hecho algo”*   5. *“Pues que haya entrado en un sitio y que tenga ese virus” “Porque se ha contagiado”*   6. *“Te pones malito porque en el suelo hay virus cuando lo pisas” “Ha ido a ver a su padre al hospital, y él también tenía cáncer, y le ha pegado un virus”*   7. *“Ha nacido enfermo, mal” “Ha nacido así”*   8. *“Que haya tomado agua sucia” “Estar en una central nuclear”*   9. *“Pasa así por el azar” “Sale de repente”* |
| 1. **Severidad**   *¿Es grave lo que tiene Jorge?* | 1. Severidad    1. Muy grave    2. Grave    3. Poco grave | - 1. *“Es muy grave lo que tiene” “Es muy grave lo que le pasa”*   2. *“Es grave” “Si, es grave”*   3. *“No es muy grave” “Es poco grave”* |
| 1. **Pronóstico**   *¿Crees que se va a curar?* | 1. Pronóstico    1. Mortal    2. Curable | - 1. *SIN RESPUESTAS*   2. *“Se va a curar” “Si, se curará”* |
| 1. **Tiempo de curación**   *¿Cuánto tiempo crees que necesita Jorge para ponerse bien?*  *¿Crees que todas las personas necesitan el mismo tiempo para ponerse bien?* | 1. Tiempo de curación    1. Días/ Semanas    2. Meses/ Años | - 1. *“Tarda en curarse unas semanas” “Se curará en unos días”*   2. *“Se pondrá bien en unos meses” “Un año y algo, y unos seis meses”* |
| 1. **Emociones**   *¿Cómo crees que se siente Jorge?*  *¿Crees que los demás niños se pueden sentir igual que él?* | 1. Emociones y sentimientos:    1. Miedo    2. Tristeza    3. Alegría    4. Dolor (físico)    5. Aburrimiento    6. Enfado    7. Soledad    8. Vergüenza    9. Preocupación/ ansiedad | - 1. *“Siente miedo” “Le da miedo”*   2. *“Triste” “Está triste por el cáncer”*   3. *“Positivo, creyendo que va a salir adelante” “Contento por ver cómo mejora cada día” “Está alegre”*   4. *“Le duele mucho” “Dolorido”*   5. *“Está aburrido” “Aburrido en el hospital”*   6. *“Está enfadado” “Siente enfado”*   7. *“Se siente solo”*   8. *“Avergonzado” “Siente vergüenza”*   9. *“Está preocupado” “Muy agobiado los primeros días”* |
| 1. **Factores asociados a emociones negativas**   *¿Qué les hace sentir peor?* | 1. Factores asociados a emociones negativas:    1. Sensaciones físicas (dolor, malestar, pérdida de pelo, hinchazón)    2. Ausencia de la familia    3. No acudir al colegio    4. Falta de relaciones sociales y amigos    5. Pensamientos negativos    6. Inactividad/ ambiente hospitalario    7. Actitud del compañero de habitación    8. Sin respuesta | - 1. *“La enfermedad. Yo estuve con unos dolores de cabeza que no podía ni soportarlos yo” “Las quimios, les revuelven mucho y les hacen estar malitos”*   2. *“No puede ver a su familia” “Que se tenga que ir su familia”*   3. *“A mí lo que más me importaba al principio era los estudios” “No es lo mismo que antes porque antes tú vas al colegio”*   4. *“Si se siente mal y no le cae bien nadie allí” “No puedes ver a tus amigos”*   5. *“Cuando se encierra en su mundo, y ve que no hay salida” “Cuando tiene momentos de pensar”*   6. *“No tiene una vida como la que él tenía” “No puedes salir”*   7. *“Por la actitud del compañero de habitación”*   8. *“No sé”* |
| 1. **Actividades que mejoran el estado emocional**   *¿Crees que puede hacer algo para sentirse mejor?* | 1. Actividades que mejoran el estado emocional:    1. Visitas    2. Juegos    3. Relaciones sociales/ amigos en el hospital    4. Adaptación al ambiente hospitalario    5. Acudir al colegio del hospital o realizar actividades académicas    6. No sabe/ no contesta | - 1. *“Pues que venga gente a visitarle” “Que estén allí con su familia”*   2. *“Los mismos juegos con los que juegan en casa” “Salir a jugar”*   3. *“Intentar ayudarle a hacer amigos” “Que sus amigos le animen”*   4. *“Intentar que se adapte un poquito más al hospital”*   5. *“Pues te pones a hacer deberes y ya se te olvida todo” “También el cole también te ayuda a olvidar todo esto de los médicos, tú estás en clase tranquilo”*   6. *”No sé”* |
| 1. **Comunicación de estado emocional**   *¿Crees que sería bueno poder hablar con alguien sobre cómo se siente?* | 1. Comunicación de estado emocional:    1. Si    2. No |  |
| 1. **A quién comunican**   *¿Con quién?* | 1. A quién comunican    1. Padres y familiares    2. Amigos    3. Personal sanitario    4. Psicólogos    5. Otros niños/ personas que hayan pasado por la misma experiencia | - 1. *“A sus padres” “Con sus papás, sus primos, sus familiares”*   2. *“A sus amigos” “A los amigos del cole”*   3. *“Se lo puede decir a las enfermeras” “A los médicos y enfermeras”*   4. *“A un psicólogo” “A la psicóloga del hospital”*   5. *“A otros niños que hayan tenido lo mismo” “A otros niños que hayan tenido cáncer”* |

Coding forms

Interview 1: Mothers of children with cancer

| Categoría primaria | Categoría secundaria | Presencia/ Si | Ausencia/ No |
| --- | --- | --- | --- |
| 1. **Reacción emocional ante el pronóstico** | Miedo | 1 | 0 |
|  | Enfado/ rabia |  |  |
|  | Tristeza |  |  |
|  | Frustración/ impotencia |  |  |
|  | Ansiedad/ angustia |  |  |
|  | Culpa |  |  |
| 1. **Comunicación con el niño respecto a la enfermedad** | Directa y honesta |  |  |
|  | Matizan la enfermedad |  |  |
|  | Ocultan la enfermedad |  |  |
| 1. **Atribución causal del cáncer** | No sabe la respuesta |  |  |
|  | Causa externa-religión |  |  |
|  | Causa interna-factores físicos |  |  |
|  | Aleatorio |  |  |
|  | Genético |  |  |

**Interview 2: children with cancer**

| **Categoría primaria** | Categoría secundaria | Presencia/ Si | Ausencia/ No |
| --- | --- | --- | --- |
| 1. **Identificación de la enfermedad** | Cáncer |  |  |
|  | Otras enfermedades |  |  |
| 1. **Atribución causal del cáncer** | Anecdótica/ sin respuesta |  |  |
|  | Conducta imprudente puntual/ Higiene |  |  |
|  | Alimentación |  |  |
|  | Golpe |  |  |
|  | Contagio |  |  |
|  | Virus-Bichos |  |  |
|  | Predisposición |  |  |
|  | Contaminación relacionada con el medio |  |  |
|  | Aleatoria |  |  |
| 1. **Severidad** | Muy grave |  |  |
|  | Grave |  |  |
|  | Poco grave |  |  |
| 1. **Pronóstico** | Mortal |  |  |
|  | Curable |  |  |
| 1. **Tiempo de curación** | Días/ Semanas |  |  |
|  | Meses/ Años |  |  |
| 1. **Emociones** | Miedo |  |  |
|  | Tristeza |  |  |
|  | Alegría |  |  |
|  | Dolor (físico) |  |  |
|  | Aburrimiento |  |  |
|  | Enfado |  |  |
|  | Soledad |  |  |
|  | Vergüenza |  |  |
|  | Preocupación/ ansiedad |  |  |
| 1. **Factores asociados a emociones negativas** | Sensaciones físicas (dolor, malestar, pérdida de pelo, hinchazón) |  |  |
|  | Ausencia de la familia |  |  |
|  | No acudir al colegio |  |  |
|  | Falta de relaciones sociales y amigos |  |  |
|  | Pensamientos negativos |  |  |
|  | Inactividad/ ambiente hospitalario |  |  |
|  | Actitud del compañero de habitación |  |  |
|  | Sin respuesta |  |  |
| 1. **Actividades que mejoran el estado emocional** | Visitas |  |  |
|  | Juegos |  |  |
|  | Relaciones sociales/ amigos en el hospital |  |  |
|  | Adaptación al ambiente hospitalario |  |  |
|  | Acudir al colegio del hospital o realizar actividades académicas |  |  |
|  | No sabe/ no contesta |  |  |
| 1. **Comunicación de estado emocional** | Si |  |  |
|  | No |  |  |
| 1. **A quién comunican** | Padres y familiares |  |  |
|  | Amigos |  |  |
|  | Personal sanitario |  |  |
|  | Psicólogos |  |  |
|  | Otros niños/ personas que hayan pasado por la misma experiencia |  |  |
